# Proportions of CD4 test results indicating advanced HIV disease remain consistently high at primary health care facilities across four high HIV burden countries

**DOI:** 10.1371/journal.pone.0226987

**Published:** 2020-01-07

**Authors:** Katherine Lamp, Seth McGovern, Youyi Fong, Charles Diko Atem, Jean Bosco Elat Nfetam, Divine Nzuobontane, Timothy Bollinger, Ilesh Jani, Nadia Sitoe, Charles Kiyaga, George Senyama, Phibeon M. Mangwendeza, Sekesai Mtapuri-Zinyowera, Naoko Doi, Trevor Peter, Jilian A. Sacks, Lara Vojnov

**Affiliations:** 1 Clinton Health Access Initiative, Boston, MA, United States of America; 2 Fred Hutchinson Cancer Research Center, Seattle, WA, United States of America; 3 Clinton Health Access Initiative, Yaoundé, Cameroon; 4 National AIDS Control Committee, Yaoundé, Cameroon; 5 Clinton Health Access Initiative, Maputo, Mozambique; 6 Instituto Nacional de Saúde, Maputo, Mozambique; 7 Central Public Health Laboratory, Kampala, Uganda; 8 Clinton Health Access Initiative, Kampala, Uganda; 9 Clinton Health Access Initiative, Harare, Zimbabwe; 10 Ministry of Health and Child Care, Harare, Zimbabwe; University of Ghana College of Health Sciences, GHANA

## Abstract

**Background:**

Globally, nearly 22 million HIV-infected patients are currently accessing antiretroviral treatment; however, almost one million people living with HIV died of AIDS-related illnesses in 2018. Advanced HIV disease remains a significant issue to curb HIV-related mortality.

**Methods:**

We analyzed 864,389 CD4 testing records collected by 1,016 Alere Pima Analyzers implemented at a variety of facilities, including peripheral facilities, between January 2012 and December 2016 across four countries in sub-Saharan Africa. Routinely collected data and programmatic records were used to analyze the median CD4 counts and proportions of patients with advanced HIV disease by country, facility type, and year.

**Results:**

Median CD4 counts were between 409–444 cells/ul each year since 2012 with a median in 2016 of 444 cells/ul (n = 319,829). The proportion of test results returning CD4 counts above 500 cells/ul has increased slowly each year with 41.8% (95% CI: 41.6–41.9%) of tests having a CD4 count above 500 cells/ul in 2016. Median CD4 counts were similar across facility types. The proportion of test results indicating advanced HIV disease has remained fairly consistent: 19.4% (95% CI: 18.8–20.1%) in 2012 compared to 16.1% (95% CI: 16.0–16.3%) in 2016. The proportion of test results indicating advanced HIV disease annually ranged from 14.5% in Uganda to 29.8% in Cameroon. 6.9% (95% CI: 6.8–7.0%) of test results showed very advanced HIV disease (CD4<100 cells/ul) in 2016.

**Conclusions:**

The proportion of CD4 test results indicating advanced disease was relatively high and consistent across four high HIV burden countries.

## Introduction

The role of CD4 testing has changed in recent years. Due to funding constraints, CD4 testing was initially recommended to ensure the sickest patients were prioritized for antiretroviral therapy and as a tool to monitor treatment [1,2]; however, recent guidelines now strongly recommend treating all HIV-infected patients, irrespective of CD4 count, and using viral load to monitor treatment efficacy [2,3]. Approximately 84% of low and middle income countries (LMIC) have adopted the WHO *Treat All* policy, but only 66% of LMIC have implemented *Treat All* into practice [4]. Further, approximately 60% of LMIC have now implemented viral load testing as the preferred treatment monitoring tool [4]. Unfortunately, access to viral load testing remains low: outside of Kenya, South Africa, and Uganda, only 47% of patients in 2017 had access to viral load testing [5]. Therefore, until *Treat All* and viral load testing are more widely accessible, CD4 testing may continue to be used to support treatment initiation eligibility and treatment monitoring [3].

As the *Treat All* and viral load recommendations are further implemented, CD4 still plays an important role in immunological and clinical management of HIV-infected patients. CD4 is recommended to monitor patients at risk for opportunistic infections and to identify patients with advanced HIV disease [3,6,7]. Though clinical acumen can be used to identify patients with advanced HIV disease–defined as patients with a CD4 <200 cells/mm^3^ or WHO clinical stage 3 or 4 disease [6]–it has been found to be poorly accurate: up to 50% of patients with advanced disease may be asymptomatic (WHO clinical stage 1 or 2) and thus missed by clinical acumen alone [8].

Nearly one million HIV-infected patients living with HIV die annually [9]. Tuberculosis and cryptococcal meningitis are the leading causes of HIV-related mortality [10,11]. Patients with advanced HIV disease, however, can have one of a number of other opportunistic infections, including pneumocystis pneumonia, etc. [10–14]. Most of these diseases can be prevented through prophylaxis or treated. Therefore, rapid and early identification of patients with advanced HIV disease, primarily through CD4 testing, in order to provide a specific package of care will be critical to reducing HIV-associated morbidity and mortality and the burden of disease [6,13,15].

Access to CD4 testing has increased over the past five years with over 19 million CD4 tests being conducted in 2017 [5]. Unfortunately, waning donor support may lead to reductions in testing coverage. However, significant capital investment in CD4 testing has already been made. In 2013, just over 4,000 conventional CD4 technologies and nearly 2,000 point-of-care technologies had been procured [16], allowing for an approximately 125 million test capacity, far exceeding the demand and need for CD4 testing [5].

Using stored testing data from point-of-care CD4 technologies placed in primary health care facilities across four high burden sub-Saharan African countries, we sought to better understand the trends and proportions of test results indicating advanced HIV disease.

## Methods

We conducted a retrospective, observational, cross-sectional analysis of routine testing data from four countries in sub-Saharan Africa (Cameroon, Mozambique, Uganda and Zimbabwe) using the Alere Pima Analyzer. Testing data captured between January 2012 and August 2016 were included for analysis, however start dates varied by country based on when the Alere Pima Analyzer was introduced nationally. This study was approved by each country’s Institutional Review Boards: Cameroon National Ethical Committee of Research for Humans, Mozambique National Health Bioethics Committee, Uganda’s Makerere University Institute of Public Health Higher Degrees, Research and Ethics Committee, Medical Research Council of Zimbabwe as well as the US-based Advarra IRB. The IRBs waived the requirement for informed consent, while all data were fully anonymized before accessed and analyzed.

The Alere Pima Analyzer automatically records data about the device, test date and time, and numerical CD4 test outcome. Additional information can be added in free text fields for the operator and/or patient identification. The Alere Pima Analyzer has the capability to transmit data collected by the device wirelessly via the cellular SMS/GPRS network to a centralized database. The majority of the data used in this analysis was transmitted via cellular network; however some records may have been extracted manually from the analyzers using a flash drive and uploaded directly to the database for facilities with poor network coverage. Once transferred, the testing data can be stored either on the device manufacturer’s proprietary data hosting and visualization service, Data Point, or on a local database. In Cameroon, Mozambique and Uganda the Pima testing data was transmitted to Data Point, and in Zimbabwe it was transmitted to a government-owned, local server and dashboard. From either type of database, the raw testing data can be downloaded into a CSV file for analysis. Testing records were provided from each country’s central POC CD4 database with each record including the following variables: unique device ID number, assay (CD4 test or control bead), CD4 count (if successful), invalid message (if encountered), coded invalid number (if encountered), operator name (if available), result date, test start and end times, internal quality control (IQC) checks, test ID number, and software version.

Device identifications were matched with programmatic records to assign the facility name, district, and region where the device was located if this was not already included in the national database. The types of facilities were categorized as hospitals, medical centers, health centers, clinics, other (prisons or laboratories), and unknown (devices that did not have a facility listed in database or programmatic records, or the facility type could not be discerned from the given available name). Any patient with a valid CD4 test was included in the analysis, irrespective of treatment experience as patient information was not included in the data capture. Advanced HIV disease was defined as having a CD4 test result < 200 cells/mm^3^, while very advanced HIV disease was defined as having a CD4 < 100 cells/mm^3^. Using these data, trends in medians and proportions of test results showing advanced and very advanced HIV disease were calculated overall, by country, and across years.

Statistical analysis was performed by applying quantile regressions with bootstrap standard errors (200 bootstrap replicates) for clustering effect of facilities within each country and implemented using the R quantreg package [17]. Ninety-five percent confidence intervals of the medians were computed based on quantiles of the binomial distribution using the R package asbio. The analysis was conducted using Microsoft® Excel® 2010 v.14.0.7183.5000 (Redmond Washington) and the R statistical programming language and environment.

## Results

Between 2012–2016, there were 1,016 Alere Pima POC CD4 devices placed at 860 facilities across Cameroon, Mozambique, Uganda, and Zimbabwe. Uganda had the most devices (48.0%) and facilities using POC CD4 (50.2%). A total of 864,389 CD4 tests were successfully performed during the time period. Invalid and internal quality control tests were excluded from this analysis (7.7% of total CD4 tests conducted).

**Fig 1:**
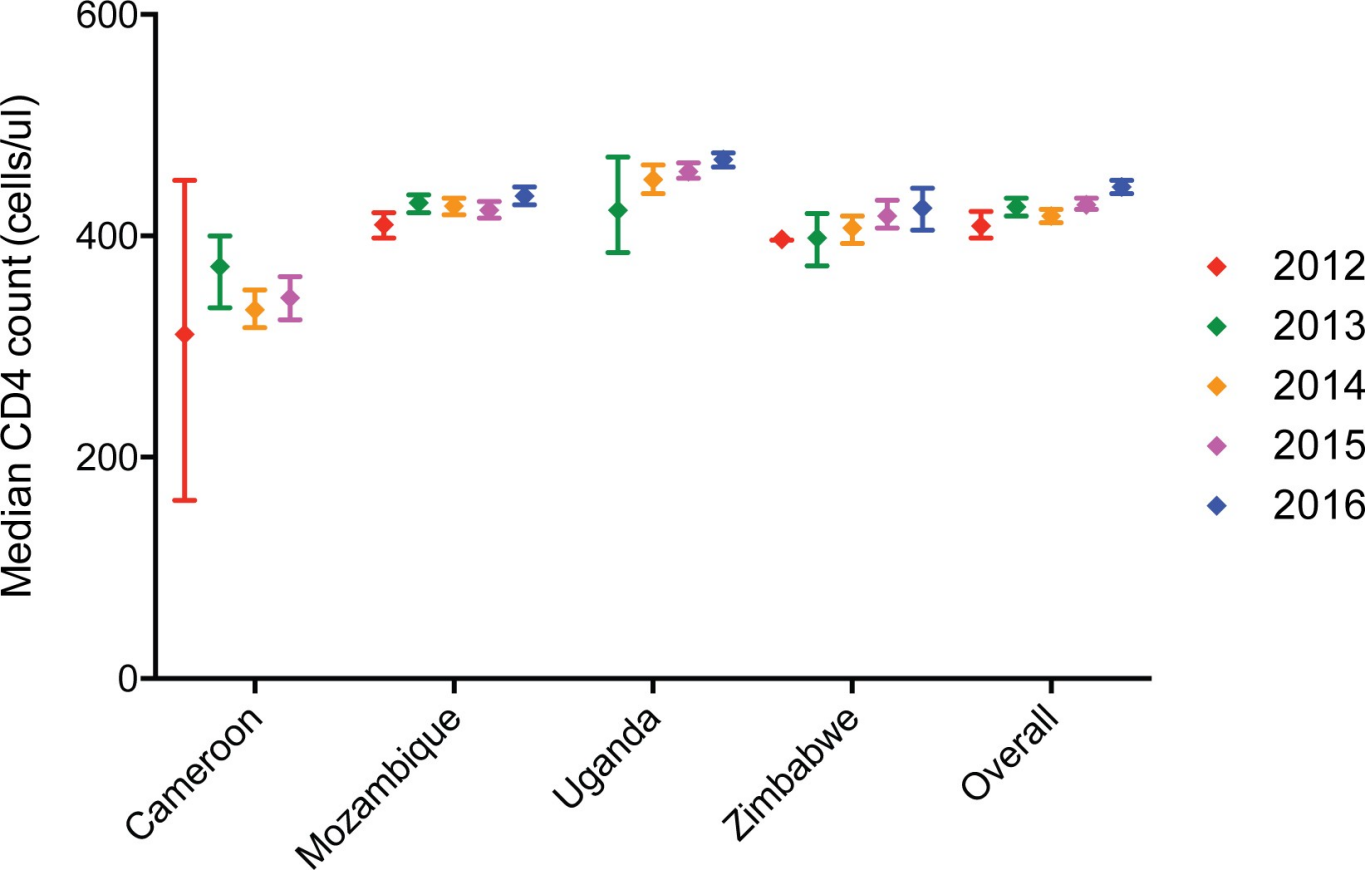
Median CD4 cell counts by year and country.

The median CD4 count in 2012 was 409 cells/ul (95% CI: 398 – 422cells/ul) and increased to 444 cells/ul (95% CI: 438–450 cells/ul) (p = 0.000) in 2016 (Table 1 and Fig 1). The median CD4 count was less than 500 cells/ul in all countries and across years. In 2016, Zimbabwe had the lowest median CD4 count of 425 cells/ul (95% CI: 402–446 cells/ul), while Uganda had the highest median CD4 count of 469 cells/ul (95% CI: 463–474 cells/ul). In Cameroon, data from 2016 were inaccessible; however, the median CD4 count in 2015 was 344 cells/ul (95% CI: 324–363 cells/ul). For most countries, the median CD4 count remained relatively stable across the study period. Median CD4 counts did not significantly increase between 2012 and 2015 in Cameroon, between 2013 and 2015 in Uganda, and between 2012 and 2014 in Zimbabwe. Significant differences were observed in Uganda between 2013 and 2016, and in Zimbabwe between 2012 and 2015/2016, although the absolute change in CD4 count over these time periods were just 46 cells/ul and 28 cells/ul respectively.

**Table 1:** Summary of CD4 results per country per year.

	n	Median (95% CI)	p-value
**Cameroon**	** **		
*2012*	180	311 (161–450)	Ref
*2013*	5,418	372 (335–400)	0.427
*2014*	16,861	333 (317–351)	0.784
*2015*	16,047	344 (324–363)	0.682
*2016*	-	-	-
**Mozambique**	** **		
*2012*	15,628	410 (398–424)	Ref
*2013*	84,891	430 (421–437)	0.003
*2014*	98,177	427 (419–434)	0.012
*2015*	161,266	423 (416–431)	0.065
*2016*	241,231	436 (428–444)	<0.001
**Uganda**	** **		
*2012*	-	-	-
*2013*	2,212	423 (385–471)	Ref
*2014*	20,092	451 (437–464)	0.136
*2015*	77,883	458 (452–465)	0.113
*2016*	74,975	469 (463–474)	0.041
**Zimbabwe**	** **		
*2012*	17	397 (396–397)	Ref
*2013*	827	398 (374–417)	0.928
*2014*	16,848	407 (393–418)	0.149
*2015*	28,213	418 (407–431)	0.003
*2016*	3,623	425 (402–446)	0.016
**Overall**	** **		
*2012*	15,825	409 (398–422)	Ref
*2013*	93,348	426 (418–434)	0.006
*2014*	151,978	418 (412–424)	0.146
*2015*	283,409	428 (424–434)	0.002
*2016*	319,829	444 (438–450)	<0.001

The median CD4 count across facility types remained below 500 cells/ul for all countries (Table 2). Overall, clinics and hospitals had the lowest median CD4 counts with 412.5 cells/ul (95% CI: 407–418 cells/ul) and 416 cells/ul (95% CI: 414–418 cells/ul), respectively. Though still below 450 cells/ul, health centers had significantly higher median CD4 counts with 435 cells/ul (95% CI: 434–436 cells/ul) than hospitals.

**Table 2:** Median CD4 counts by facility type per country.

	n	% of Total	Median (95% CI)	p-value
**Cameroon**	** **			
*Hospital*	21,572	56.0%	342 (337–346)	Ref
*Medical Center*	3,551	9.2%	348 (337–358)	0.358
*Health Center*	3,878	10.1%	351.5 (341–362)	0.153
*Clinic*	134	0.3%	436.5 (381–516)	0.003
*Unknown*	9,371	24.3%	338 (331–345)	0.376
*Other*	-	-	-	-
**Mozambique**	** **			
*Hospital*	70,121	11.7%	430 (428–432)	Ref
*Medical Center*	-	-	-	-
*Health Center*	481,352	80.1%	428 (427–429)	0.206
*Clinic*	19,286	3.2%	457 (453–463)	<0.001
*Unknown*	30,434	5.1%	436 (432–440)	0.022
*Other*	-	-	-	-
**Uganda**	** **			
*Hospital*	33,464	19.1%	433 (430–437)	Ref
*Medical Center*	-	-	-	-
*Health Center*	130,231	74.3%	468 (466–469)	<0.001
*Clinic*	990	0.6%	422.5 (399–439)	0.373
*Unknown*	9,914	5.7%	479 (473–485)	<0.001
*Other*	563	0.3%	478 (454–505)	0.002
**Zimbabwe**	** **			
*Hospital*	12,944	26.1%	410 (404–416)	Ref
*Medical Center*	-	-	-	-
*Health Center*	24,668	49.8%	416 (412–420)	0.125
*Clinic*	11,012	22.2%	411 (405–418)	0.827
*Unknown*	-	-	-	-
*Other*	904	1.8%	457.5 (430–476)	<0.001
**Overall**	** **			
*Hospital*	138,101	16.0%	416 (414–418)	Ref
*Medical Center*	3,551	0.4%	348 (337–358)	<0.001
*Health Center*	640,129	74.0%	435 (434–436)	<0.001
*Clinic*	31,422	1.4%	412.5 (407–418)	0.382
*Unknown*	49,719	5.8%	426 (423–430)	<0.001
*Other*	1,467	0.2%	466 (451–481)	<0.001

**Fig 2:**
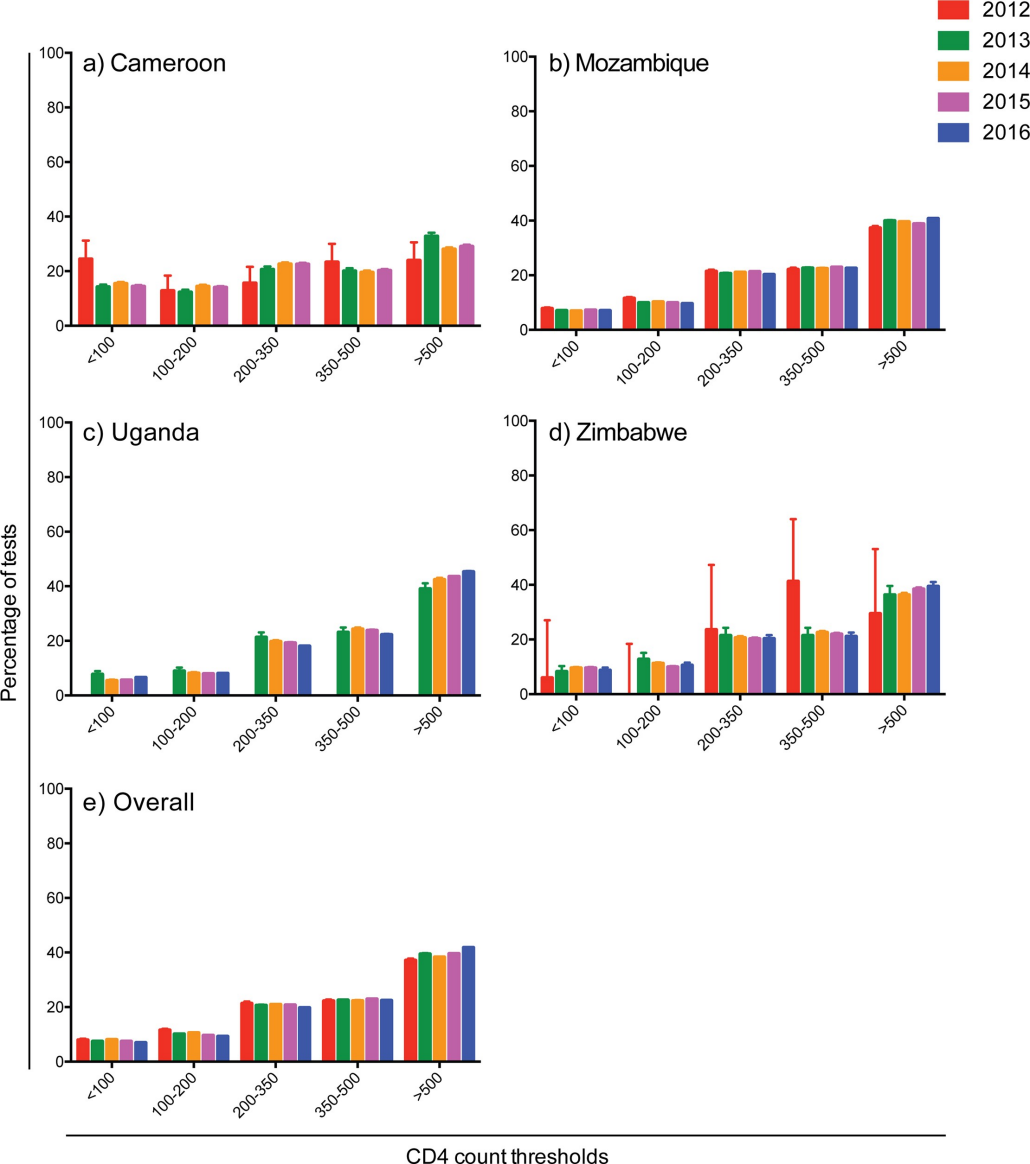
Percentage of tests with CD4 cell count thresholds by year and country.

The overall proportion of test results with a CD4 count above 500 cells/ul increased from 37.1% (95% CI: 36.3–37.8%) in 2012 to 41.8% (95% CI: 41.6–41.9%) in 2016 (Fig 2). Similar increases were observed across countries. However, it is notable that the proportion of test results with a CD4 count below 100 cells/ul (very advanced HIV disease) or between 100–200 cells/ul have not correspondingly decreased considerably. In 2012, 7.9% (95% CI: 7.5–8.4%) of all test results had a CD4 count < 100 cells/ul, while 6.9% (95% CI: 6.8–7.0%) did in 2016. Further, 11.5% (95% CI: 11.0–12.0%) had a CD4 count between 100–200 cells/ul in 2012, while 9.2% (95% CI: 9.1% - 9.3%) had a CD4 count between 100–200 cells/ul in 2016. The proportion of test results indicating advanced HIV disease (< 200 cells/ul) was 19.4% (95% CI: 18.8–20.1%) in 2012 and 16.1% (95% CI: 16.0–16.3%) in 2016 (Table 3). Though the proportion has decreased between 2012 and 2016, the trend has remained relatively stable over the years analyzed.

**Table 3:** Proportion of patients with advanced HIV disease (< 200 cells/ul).

	2012	2013	2014	2015	2016
< 200 cell/ul	19.4% (18.8–20.1)	17.5% (17.2–17.7)	18.5% (18.3–18.7)	17.0% (16.8–17.1)	16.1% (16.0–16.3)
*> 200 cells/ul*	80.6% (79.9–81.2)	82.5% (82.3–82.8)	81.5% (81.3–81.7)	83.0% (82.9–83.2)	83.% (83.7–84.0)

The overall proportion of test results across years indicating advanced HIV disease was highest in Cameroon with 28.8% and lowest in Uganda with 14.0%. In the final year of available data, Cameroon had a proportion of test results indicating advanced HIV disease of 28.3% (2015), Mozambique had 16.6% (2016), Uganda had 14.5% (2016), and Zimbabwe had 19.2% (2016).

Finally, we looked at the proportions of test results indicating advanced HIV disease by facility type. In hospitals, the proportion of test results indicating advanced HIV disease was between 17–22% per year and 17.2% (95% CI: 16.8–17.6%) in 2016. In health centers, the proportion of test results indicating advanced HIV disease was between 16–20% per year and 16.1% (95% CI: 16.0–16.3%) in 2016. In medical centers, the proportion of test results indicating advanced HIV disease was between 12–21% and 12.7% (95% CI: 11.9–13.5%) in 2016. In clinics, the proportions of test results indicating advanced HIV disease was between 15–21% and 15.2% (95% CI: 13.3–17.2%) in 2016.

## Discussion

This analysis was the first of its kind in national, publicly-supported health care facilities outside of South Africa and the first looking at decentralized settings with point-of-care technologies. Even though treatment eligibility thresholds increased during the study period from 350 cells/ul to 500 cells/ul and finally to treating all patients with HIV irrespective of CD4 count [1–3], the median CD4 count remained below 500 cells/ul across all years and countries. Further, over the study period 147,215 out of 864,389 (17.0%) CD4 results were < 200 cells/ul. In 2016, 16.1% of test results indicated advanced HIV disease and 6.9% showed very advanced HIV disease.

Several recent studies have highlighted the continued significant burden of advanced HIV disease. In 2016 in South Africa, for example, nearly a third (32.9%) of over 650,000 HIV-positive patients had advanced HIV disease at the time of their first CD4 count prior to ART initiation [18]. Further, reviewing CD4 data in nearly one million patients attending health care facilities within the IeDEA and COHERE cohorts across 55 countries found that not only has the proportion of patients entering care remained high across settings (31% in low-income countries, 40% in low- and middle-income countries, 29% in high-income countries), but these rates have plateaued over the past few years [19]. Interestingly, many patients entering, or re-entering care, are treatment experienced [20,21]. In South Africa, 56.7% of patients entering care in 2017 with very advanced HIV disease were treatment experienced (20). The prevalence of advanced HIV disease was lower in the current study than reported in the literature [18–21]; however, this could have been due to the variable uses of CD4 testing across included countries within the current study. During the study period, CD4 was still used to monitor treatment as access to viral load testing was significantly below coverage rates in South Africa [5].

It is sometimes suggested that the majority of advanced HIV disease cases are likely to present at centralized hospital settings; however, the results presented here highlight the challenge and prevalence of advanced HIV disease in smaller, rural, and/or decentralized primary settings. Health centers and clinics, for example, had proportions of advanced disease of 15% and 16%, respectively, in 2016 with historic proportions above 20% for certain years. Similar to the REALITY trial that showed significant proportions of patients with advanced disease at outpatient wards [8], our data suggests that primary care facilities have high proportions of patients with advanced HIV disease that also require the advanced HIV disease package of care in order to minimize HIV-related morbidity and mortality [6].

Patients with advanced HIV disease are at increased risk of HIV-related morbidity and mortality–tuberculosis and cryptococcal meningitis are the leading causes of mortality [8,14,21,22]. Rapid and early identification of patients with advanced HIV disease will be critical to reduce the risk of death. Since nearly half of all patients with advanced HIV disease are asymptomatic [8], CD4 testing will remain necessary to more accurately diagnose those with advanced HIV disease and at high risk for HIV-related morbidity and mortality. Once identified and linked, it is recommended that patients with advanced HIV disease receive a package of care, including rapid ART initiation, screening for tuberculosis and cryptococcal meningitis, preemptive fluconazole treatment as well as cotrimoxazole and isoniazid prophylaxis [6].

Due to the high risks of imminent morbidity and mortality for people living with HIV identified with advanced HIV disease, point-of-care CD4 testing that allows same-day result return provides a novel solution to ensure rapid test result return, linkage, and clinical decision-making. Several device-based point-of-care CD4 technologies currently exist to support rapid identification and linkage to advanced HIV disease care with rapid, device-free CD4 tests in development [23]. However, in lieu of a market-ready rapid lateral flow or dipstick CD4 test, the significant investment already made for both laboratory-based and device-based point-of-care CD4 technologies should allow for considerable access to CD4 testing to support identification of patients with advanced HIV disease across the health care system.

While optimized antiretrovirals, including dolutegravir, have become the preferred first line treatment in several high burden countries [5,24] and exhibit lower toxicity and a higher barrier to drug resistance, additional factors, such as stigma and discrimination, are unlikely to be affected by their introduction. As long as these critical societal issues remain, challenges in finding and retaining patients in care will persist, as well as associated HIV-related morbidity and mortality.

Several limitations exist in this study. First, due to a lack of unique patient identifiers it was not possible to decipher distinct patients and some repeat testing may be included. Second, we were unable to differentiate between test results for CD4 testing conducted at baseline to determine treatment eligibility and those for monitoring patients already on treatment. Further, for those patients tested for treatment eligibility, it was not possible to discern between those with and without prior treatment experience. Additionally, patient selection for CD4 testing at all health care facilities relied solely on national guidelines and/or clinical acumen; therefore, there was a risk of targeted patient selection for testing. However, advanced HIV disease can affect patients at any time and whether treatment experienced or not–all test results indicating advanced HIV disease should be clinically acted upon to better manage and support those sick patients. The advanced HIV disease package of care as recommended by the WHO should be implemented for all patients identified with advanced HIV disease, regardless of treatment experience and time of identification. Furthermore, while it is expected that sick patients are more likely to be CD4 tested repeatedly, the data here provide a cross-sectional snapshot of the proportion of test results indicating patients with advanced HIV disease and in need of the package of care to minimize morbidity and mortality. Thus, these findings are still valuable for providing a national public health perspective and national proportions of patients with advanced HIV disease. Third, data were derived from health care facilities with point-of-care CD4 technologies, which were generally more decentralized and/or rural and/or within primary health care facilities; CD4 test results from larger facilities that rely on conventional, laboratory-based CD4 are not reflected in this analysis, primarily because these laboratory-based technologies do not have similar connectivity capabilities. Finally, though p-values indicated significant differences across some years and facility types, the sample size included within the programmatic review study were very high, where small, likely non-clinically relevant differences may have shown statistical significance. The results within, and suggested differences, should be interpreted within such context.

The proportion of CD4 test results indicating advanced HIV disease was relatively high and consistent across four high HIV burden countries, despite increases in treatment coverage and adoption of Treat All policies during the time period. CD4 testing supports identification of patients with advanced HIV disease in need of follow-up care and a suggested package of interventions in order to minimize HIV-related morbidity and mortality. Management for patients identified with advanced HIV disease, including in peripheral facilities and using point-of-care technologies to support faster identification, should be a priority in order to support sick patients and reduce HIV-related morbidity and mortality.
